# Electromagnetic Performance Characterization and Circuit-Level Modeling of a Miniaturized Meander-Line Antenna for Implantable and Wearable RFID Applications

**DOI:** 10.3390/s26061744

**Published:** 2026-03-10

**Authors:** Waqas Ali, N. Nizam-Uddin, Ubaid Ullah, Muhammad Zahid, Sultan Shoaib

**Affiliations:** 1 Department of Electrical Engineering, HITEC University, Taxila 47080, Pakistan; waqas.ali@hitecuni.edu.pk (W.A.); yanizamuddin@gmail.com (N.N.-U.); ubaid.bme@gmail.com (U.U.); 2 Department of Biomedical Engineering, HITEC University, Taxila 47080, Pakistan; 3 Department of Telecommunication Engineering, University of Engineering and Technology, Taxila 47050, Pakistan; muhammad.zahid@uettaxila.edu.pk; 4 Department of Engineering, Gulf University for Science and Technology, Hawally 32093, Kuwait

**Keywords:** implantable antenna, biomedical applications, IoT, materials, implantable technology, phantom modeling, SAR analysis

## Abstract

This paper proposes a small size meander-line patch antenna which is designed to have biomedical telemetry applications using the Industrial, Scientific and Medical (ISM) band from 2.40 to 2.48 GHz supported by the equivalent circuit model (ECM). Antenna miniaturization is realized by the effective use of several slot structures placed in the rectangular microstrip patch structure, in order to realize electrical length extension and reduce the physical size. The antenna has overall dimensions of 12 × 22 × 0.787 mm^3^ and is made on a low-loss Arlon AD 450 (ε_r_ = 4.50 and tanδ = 0.0035) dielectric substrate, which has the desired stable electrical behavior and, importantly, can be used in implantable environments. Experimental validation is done by implanting the fabricated prototype into a laboratory-manufactured tissue-mimicking phantom, and it showed good agreement with simulated results. The designed antenna has a peak gain of 1.29 dBi in free space and −24.99 dBi at a frequency of 2.45 GHz and a fractional impedance bandwidth of about 250 MHz, which will guarantee reliable operation in the face of diversity and fluctuation in the surrounding environment (biological tissues). Furthermore, specific absorption rate (SAR) analysis is carried out in order to comply with international safety standards with peak SAR values kept within the permissible level of 2 W/kg for 10 g averaging tissue. The results show that the proposed antenna provides a good trade-off between the reduction in size, radiation performance and safety to the patient, making it a good candidate for short-range in-body wireless communication, implantable medical devices, and biomedical monitoring systems.

## 1. Introduction and Background

**Figure 1:**
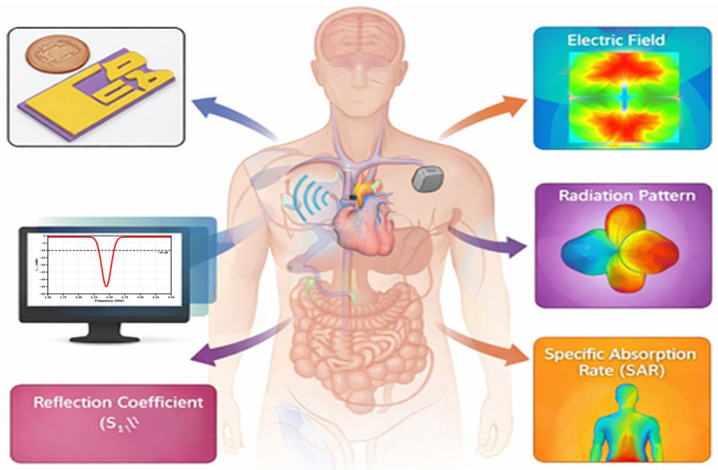
Depiction of Embedded Antenna for On-Body and In-Body Communication.

Implantable antenna technology has become a major breakthrough in biomedical applications today. The implantation of antennas has a very important role in biomedical diagnosis, therapeutic procedures and biotelemetry systems, where electromagnetic wave-based techniques are presented inside the human body. These types of antennas allow implantable medical devices (IMDs) to gather information about physiological parameters and transmit them wirelessly to outer base stations for monitoring and analysis [1,2,3]. Figure 1 shows the conceptual framework for assessing the performance of an implantable antenna within the human body, the key electromagnetic and system-level parameters involved by the evaluation. The antenna is illustrated operating in the proximity of biological tissues. Its interaction is evaluated by means of several characteristics, such as the electric field distribution, far-field radiation pattern, reflection coefficient (S11), and specific absorption rate (SAR). This type of integrated representation highlights the coupled relation between the characteristics of the antenna and the chosen environment of the anatomical site such that tissue loading affects impedance matching, the behavior of the radiation, and the cancer tumor energy absorption processes. However, such a holistic evaluation is very much essential for the design of implantable and wearable antennas for reliable wireless communication, ensuring the compliance of biological safety constraints.

Implantable antennas are necessary for facilitating wireless data transmission between devices inside the body and monitoring systems outside the body. Medical implants are extensively used in detecting, diagnosing and continuously monitoring the health condition of the patient. One of the major applications of implantable antennas is biotelemetry, and a lot of research is being devoted to develop medical implants within specified frequency bands with low interference to surrounding systems. Since the transmission of the data is done wirelessly, it is also a critical issue to ensure the security of the data and the privacy of the people wearing the IMDs [4,5,6].

The assignment of radio frequency or RF-spectrum bands used by medical implant applications differs in each region and is determined by the local regulatory body. In the United States, frequency allocations for medical implants are divided into short-range and long-range allocation by the Federal Communications Commission (FCC). In Europe, the spectrum allocation is regulated by the European Communications Committee (ECC), which differentiates between active medical implant devices and associated peripheral equipment, with spectrum ranges being specifically allocated for medical data acquisition. Compared to antenna design under the free-space condition, designing for in-body applications is a much more difficult task owing to the complex electromagnetic environment of biological tissues [7,8,9]. Table 1 shows the frequency bands for medical and implantable communication systems.

**Figure 2:**
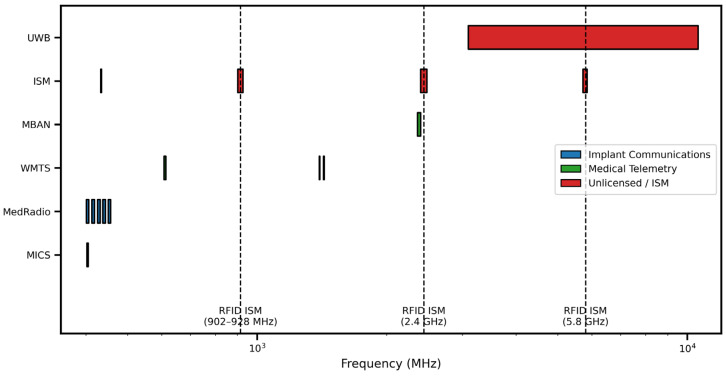
Log-scale frequency allocation of medical and RFID ISM bands.

Figure 2 shows the log-scale frequency allocation of medical and wearable wireless communication services with bands divided into implant communications, medical telemetry, and unlicensed/ISM applications. Distinct frequency segments are indicated for MICS and MedRadio in the sub-GHz range, WMTS and MBAN in the GHz range as well as broad allocations such as UWB at higher frequencies. The ISM bands in the frequency range of 902–928 MHz, 2.4 GHz and 5.8 GHz in which RFIs are relevant are explicitly highlighted, giving special importance to their use in wearable and identification systems. This representation paints an easy-to-understand picture of the spectrum segregation between regulated medical services and unregulated bands, or at least it gives a good abridged overview of the operational frequency picture for wearable, implantable, and radio frequency identification (RFID)-enabled wireless devices.

Higher operating frequencies are associated with shorter wavelengths, allowing for great reductions in the dimensions, which are the antennas. Antennas that operate in higher frequency bands also have wider bandwidths for transmitting data at higher rates and achieving better communication. However, such high frequency operation is much more affected by biological tissues, as more tissue absorption and signal attenuation happen as compared to the lower frequency bands. In the range of frequencies of 3–5 GHz, the signal is attenuated about 20–30 dB per every 2 cm of biological tissue. While lower operating frequencies have lower tissue attenuation, they inherently have a lower ability to carry communication data and require the use of bigger antennas and bulkier circuit components. These factors contribute to an increase in the size of implantable medical devices as a whole, which creates additional limitations on the ability to design miniature and comfortable devices [8,10,11].

**Figure 3:**
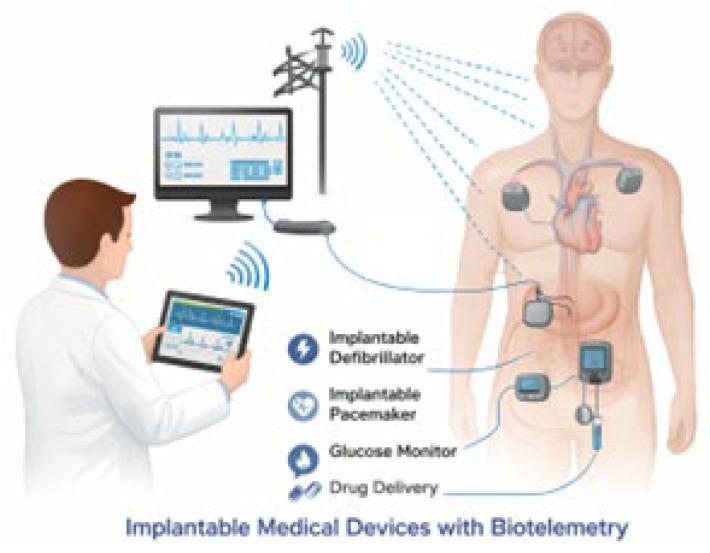
Model of an implantable medical device with biotelemetry.

Figure 3 provides a conceptual model of implantable medical devices with system biotelemetry. Various implantable devices are suitably placed in different portions of the human body to monitor some physiological parameters. Data gathered by the IMDs are sent wirelessly to some external receiver and stored in a cloud-based database, from where the information can be accessed and sent to healthcare professionals or concerned stakeholders for data monitoring and analysis.

The use of implantable medical technologies started in the 1960s, with the creation of the first biomedical implantable device, the cardiac pacemaker, designed to treat abnormal heart rhythms. Since then, the use of implantable antennas has continued to grow in a wide array of medical applications. Notably, in cancer diagnosis and treatment, implantable radiating elements are used for generating localized and controlled heating, for therapeutic purposes, in order to minimize the damage to the surrounding healthy tissues [12,13,14].

With the development of Healthcare Technology and awareness of the monitoring of one’s health, IMDs have gained a lot of acceptance and importance. Today, various implanted systems such as implantable drug delivery devices, implantable defibrillators, pacemakers, etc., are a routine part of clinical practice. Implantable antennas further help in continuous monitoring of critical physiological parameters such as blood glucose level, body temperature, and heart functions, thus allowing to provide real-time diagnostics and better patient care [15,16,17].

In recent years, there have been major breakthroughs in the development of implantable sensors that can continually monitor the physiological parameters of the patient. These devices can be implanted into the body and have to effectively communicate with external monitoring devices, which requires the incorporation of small and efficient antennas. However, the design of antennas for in-body application provides significant challenges because of the complicated electromagnetic properties of human tissues. Some of the main design considerations include high tissue conductivity, impedance matching, stringent size considerations, low power operation, and biocompatibility requirements. For proper assessment of the performance, it is necessary to perform realistic simulations using the dielectric properties and the anatomical geometry of the surrounding biological tissues in antennas [18].

Microstrip patch antennas have attracted significant interest for implantable use because of their design flexibility and conformability and ease of miniaturization. Various miniaturization techniques, e.g., meandered dipole structures, split ring resonators (SRRs) and slot-based structures, have been widely used to improve antenna performance within small physical dimensions. Comprehensive examinations of implantable antenna designs are offered in the current literature [19,20,21].

Implantable antennas are generally designed to operate within specific bands designated for frequency bands resulting in Medical Implant Communication Service (MICS) and Industrial, Scientific and Medical (ISM) bands. In this work, a novel-style microstrip patch antenna is proposed for biomedical telemetry applications working in the ISM band. Owing to their compact size and favorable electromagnetic characteristics, SRRs are incorporated to provide miniaturization and better performance. Compared to previously reported implantable antenna designs, the proposed implantable antenna has a low-loss, low-cost and small structure and is a suitable candidate for ISM-band biomedical implant applications [22,23,24,25].

Implantable antennas provide constant real-time monitoring of vital signs and physiological parameters and make the disease diagnosis possible early, enabling clinical intervention in time. By supporting wireless communication, these antennas improve patient mobility by removing the need for physical connections to external equipment used to monitor the patient, thereby enhancing the comfort and quality of life. Their use is also encouraged as minimally invasive healthcare as they enable fewer hospital visits and invasive diagnostic procedures to be performed, which also reduces the risk of infection and patient discomfort. Furthermore, implantable antennas are also making remote healthcare and telemedicine more accessible, as they make it easier for implantable medical devices to transmit data to healthcare providers reliably and thus make medical services accessible to patients in remote or underserved areas. Additionally, they are critical parts of smart implantable systems like pacemakers, insulin pumps, and neural stimulators because of their ability to provide precise control of the device, to make it programmable, and to perform real-time optimization of performance [26].

Unlike traditional antennas for functioning in free space, implantable antennas that are part of different sections of the human body must comply with stringent aesthetic and functional demands under various climatic conditions. In addition to ensuring an unobtrusive form factor, characteristics such as biocompatibility, miniaturization, patient safety, adequate far-field radiation performance, and low power consumption are important design considerations for implant-based antennas.

Biocompatibility is one of the most important considerations of designing implantable antennas, involving the direct impact of patient safety as well as reducing unwanted radiation exposure to surrounding biological tissues. Since human tissues have conductive properties, direct contact between the antenna metallization and body tissues is a possible circumstance that potentially leads to short-circuiting and the degradation of antenna performance. Therefore, avoidance of unwanted electrical contact and long-term biocompatibility are key requirements for reliable and safe implantation.

Biocompatibility can be achieved in two main ways: the introduction of either a dielectric superstrate layer or biocompatible coating. The most used technique is to use a dielectric superstrate to physically separate the metallic radiating element from the biological tissues to ensure electrical isolation and increase antenna reliability. Commonly used superstrate materials include Teflon, MACOR and ceramic alumina. Alternatively, implantable antennas can be encapsulated through thin layers of biocompatible materials like zirconia, polyether ether ketone (PEEK) and silastic MDX 4210 manufactured by Dow Corning which is based in Midland, United States that facilitate effective insulation and long-term implantation compatibility.

Miniaturization is amongst the most important design considerations for implantable antennas. Recent advances in the technologies for fabricating antenna have allowed for the design of extremely compact implantable antennas for highly constrained biomedical applications. For example, devices like cochlear implants and retinal prostheses need very small antennas which can be safely embedded into the auditory nerves and the eyeball, respectively. Conventional half- or quarter-wavelength antenna geometries having designations for medical frequency bands (such as the Medical Implant Communication Service (MICS) and Medical Device Radiocommunications Service (Med Radio)) are less than ideal for implantation because of their relatively large physical dimensions. Therefore, effective miniaturization of antennas at these frequency ranges still poses a challenging task.

The full-wave electromagnetic analysis of the proposed antenna design was done with CST Microwave Studio 2024 based on the time-domain Finite Integration Technique (FIT). The simulated performance of the antenna is analyzed such as reflection coefficient (S_11_), radiation performance, electric field distribution and specific absorption rate (SAR).

## 2. Proposed Antenna Design

**Figure 4:**
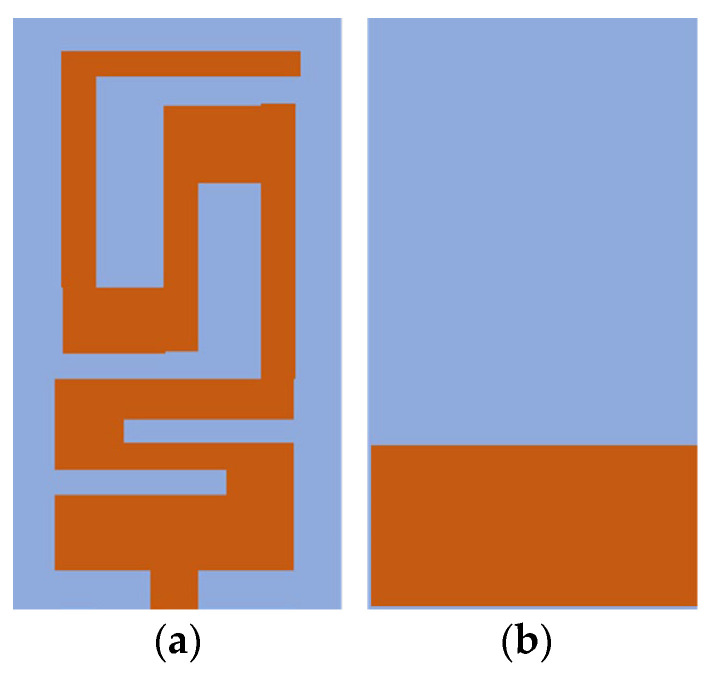
Proposed antenna geometry: (**a**) front view; (**b**) back view.

Figure 4 represents the proposed antenna geometry. The proposed antenna uses the radiating structure of a meandered microstrip, which is a common miniaturization technique for size-constrained wireless and implantable applications. By folding the current path into a serpentine geometry, the effective electrical length of the antenna is greatly increased without expanding the physical footprint of the antenna that can be used to resonate at a desired operating frequency in a limited area. The planar structure is a way to easily fabricate it on a dielectric substrate and makes it possible to integrate it with miniaturized electronic circuitry. Furthermore, in the meandered structure, there is even more induction loading to help match impedance and to tune bandwidth while keeping good radiation performance. Owing to the compact size, structural simplicity, and desired electromagnetic characteristics, the proposed meandered microstrip antenna design is ideally suited for the implantable and in-body wireless communication systems in the medically allocated frequency bands.

Table 2 shows the comparison between the proposed antenna and other state-of-the-art existing antennas in connection to size, frequency, substrate, bandwidth, gain and design technique. The proposed antenna has a positive value of realized gain, that is, 1.29 dBi in free space and −24.99 dBi inside the phantom, while negative values of gain are realized for all the previously reported compact antennas working in the same frequency band, varying from −11.8 dBi to −29 dBi. This work presents therefore a significant advance in compact antenna design since the inherent inefficiency in radiation, a usual side effect of miniaturized structures, is overcome so that practically useful radiation performance in low-profile form on these designs is possible.

**Figure 5:**
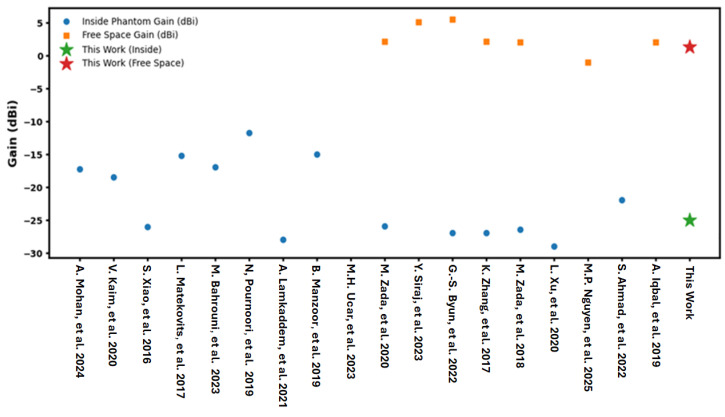
Scatter comparison of realized gain for reported antennas and proposed design in free space and surrounding environment.

Figure 5 shows the comparative gain performances of previously reported implantable antennas and the proposed design in terms of free-space and inside-phantom gain, same as reported in Table 2. It is seen that most of the compact implantable antennas generally suffer from significant degradation inside the body tissue, where the inside phantom gain is generally in the negatively prior range of around −29 dBi to −15 dBi due to the high dielectric losses and impedance detuning effects. The proposed antenna shows inside-phantom gain of −24.99 dBi, which is comparable or even improved compared to some aspects of miniaturized designs reported in the literature that are operating in the ISM band of 2.4–2.45 GHz. Additionally, the stability of the free-space gain, which is 1.29 dBi, is maintained. Notably, this performance has been reached with the limited form factor (12 × 22 × 0.787 mm^3^) and in a meander line setup, indicating the effective trade-off between size, bandwidth and radiation efficiency. Therefore, the proposed work provides competitive gain performance and maintains structural compactness, which suits biomedical implant applications where size and tissue compatibility are challenges.

### 2.1. Initiating Antenna Design

The antenna design and electromagnetic analysis were performed in CST Microwave Studio. An Arlon AD 450 substrate that has a relative permittivity of 4.50 and a dielectric loss tangent of 0.0035 was used to achieve low-loss performance. The thickness of the radiating patch was chosen as 0.035 mm in order to obtain a compact and lightweight structure. One of the characteristics of the proposed design is the introduction of a structure consisting of a meander line in a conventional rectangular microstrip patch, capable of making a longer electrical path length with a small physical size. The initial design parameters of the rectangular patch antenna are given and discussed below:

(1) $$W=\frac{v_{0}}{{2f}_{r}}\sqrt{\frac{2}{1+\epsilon_{r}}}$$

(2) $$L=\frac{v_{0}}{{2f}_{r}\sqrt{\epsilon_{reff}}}-2\Delta L$$

(3) $$\epsilon_{reff}=\frac{\epsilon_{r}+1}{2}+\frac{\epsilon_{r}-1}{2}{\left[1+10\frac{h}{W}\right]}^{-\frac{1}{2}}$$

(4) $$\Delta L=0.412h\frac{\left(\epsilon_{reff}+0.3\right)\left(\frac{W}{h}+0.264\right)}{\left(\epsilon_{reff}-0.258\right)\left(\frac{W}{h}+0.8\right)}$$

Here, W and L are the width and length of the radiating patch, respectively, while $\epsilon_{reff}\;$ represents the interrelated effective dielectric constants of the substrate. The parameter ΔL takes account of the incremental extension in the effective length of the radiating patch due to the fringing fields at the edges of the patch. In this context $f_{r}$ represents the resonance working frequency, $\epsilon_{r}$ = dielectric constant of substrate, and $v_{0}$ is the speed of light in free space. The dimensions of the ground plane for the patch antenna can be computed by means of the expressions shown in Equations (5) and (6).

(5) $$L_{g}=L+6\times h$$

(6) $$W_{g}=W+6\times h$$

Initially, rectangular patch antenna parameters were calculated by using the above-mentioned Equations (1)–(6), and the patch was simulated in CST Microwave Studio.

It should be noted that Equation (3) is used for preliminary analytical estimation of the patch dimensions on the basis of conventional microstrip antenna theory under the assumption of air superstrate. This formulation gives a good approximation of the first order of the effective permittivity and of the corresponding resonant length in the first design stage. The final antenna geometry, however, is not independent of this analytical expression and is then optimized with a full-wave electromagnetic solver. After getting the first dimensions from analytical calculations, the antenna was simulated and optimized with a full-wave electromagnetic model, setting up and taking into consideration the fringing fields, substrate properties, boundary condition, and coupling effects. The optimized antenna was first tested in free space to gain some baseline performance.

**Figure 6:**
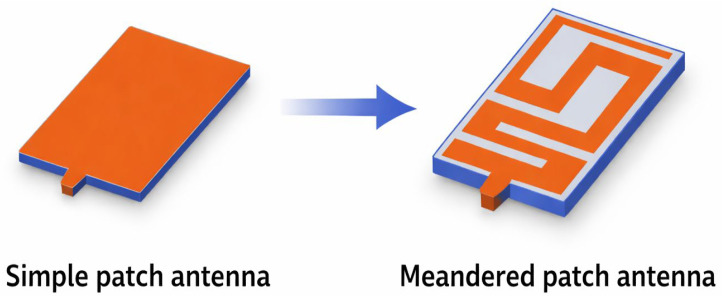
Evolution from a simple patch to a meandered antenna.

Then, rectangular slots were added to achieve the meander-line design and the required resonant frequency within the proposed band (2.40 GHz to 2.48 GHz). The final design of the patch is shown in Figure 6. 

### 2.2. Design Evolution Steps

**Figure 7:**
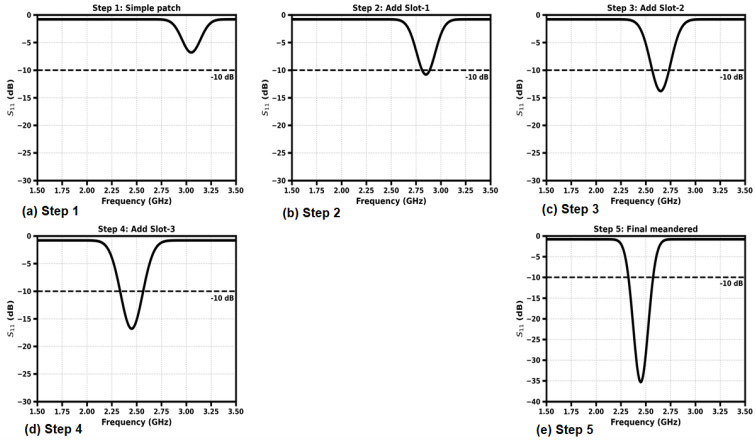
S_11_ of the design steps.

Figure 7 shows the step-wise development of the proposed implantable antenna from the standard rectangular patch shape to the final miniaturized meandered shape. In Step 1, the initial-pass full patch resonates around the 3.1 GHz region with a shallow return loss and has poor operating bandwidth. Step 2 adds the first modification of the slot that shifts the resonance down and increases the radiation path length, which leads to better impedance matching. In Step 3, there is one more slot to etch, which further lowers the resonant frequency but improves the return loss depth. Step 4 shows the effect of introduction of a third slot, which continues the process of miniaturization and allows a deeper resonance in the range of 2.5 GHz. Finally, Step 5 depicts the final complete meandered topology with good impedance matching, sharply minimized (−35 dB) return loss, and resonance close to 2.45 GHz. The gradual changes validate the value of systematic modification of slotting and folding of conductors that effectively reduces the antenna size while improving the performance for implantable application.

**Figure 8:**
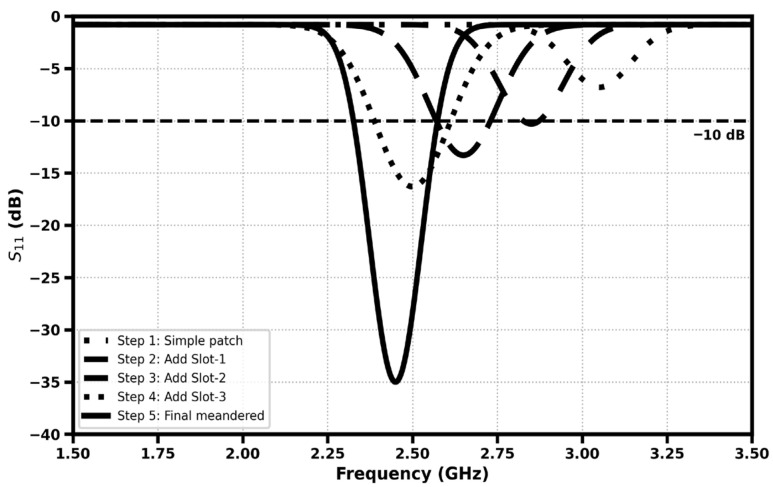
Parametric S_11_ comparison showing performance evolution.

Figure 8 shows the gradual development of the antenna design and the impact on the return loss performance. Beginning with the simple rectangular patch, subtle downward shifts of the resonance frequency are obtained with the addition of the sequential slots, and impedance matching is enhanced. Each such added slot allows for further miniaturization so as to add electrical length to the radiator. The last meandered form results in the highest resonance, which shows the highest reflection coefficient of about −35 dB around 2.45 GHz; i.e., efficient radiation and optimal matching at the ISM band is achieved.

### 2.3. Optimization of the Design

**Figure 9:**
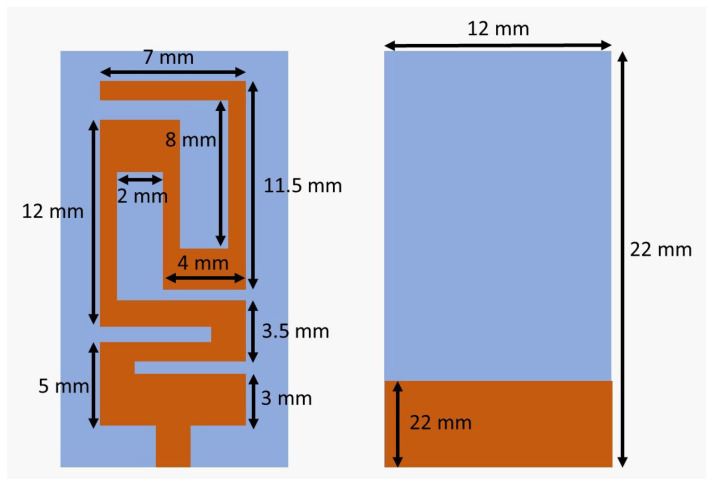
Post-optimization parameters.

The size of the proposed antenna is with a miniature footprint of 12 × 22 mm^2^, and it consists of a rectangular Arlon AD 450 substrate and a reduced-size meander radiation patch. The radiating structure combines many folded paths (including vertical trace segments of 12, 11.5 and 5 mm as well as 7, 8 and 4 mm horizontal ones, connected by 2–3.5 mm meander gaps), in order to achieve optimal electrical length within a limited physical space. The copper metallization fills the low part of the substrate, which acts as the ground plane with a size of 12 × 3 mm. This geometric configuration manages to lower the resonant frequency and improve the radiation performance with a small implantable geometrical form capable of operating in ISM-band frequencies. The design is based on a partial ground. The post-optimization dimensions are shown in Figure 9.

### 2.4. Material Description

The proposed antenna has overall dimensions of 12 × 22 × 0.787 mm^3^ built on Arlon AD 450 substrate for implantable applications. Arlon AD 450 substrate is recognized as appropriate for implantable antenna applications owing to a combination of good electromagnetic properties, high-frequency mechanical stability and reliability. Arlon materials usually have a moderate dielectric constant, to ensure the miniaturization of antennas while still having reasonable bandwidth and radiation efficiency. Their low dielectric loss tangent results in low loss in the signal, especially important for implantable antennas which are operating in lossy biological surroundings in which efficiency is already compromised. In addition, Arlon substrates offer great dimensional stability and homogeneous dielectric properties to ensure homogenous system antennas under fabrication tolerances and long-term operation. From a practical standpoint, Arlon laminates are chemically stable, moisture-resistant, and compatible with the usual PCB fabrication processes, which makes them reliable for encapsulated implantable devices. When combined with a biocompatible superstrate or coating, Arlon substrates provide a good balance between performance, manufacturability and long-term reliability for implantable in-body wireless communication systems.

## 3. Analysis of the Simulated and Experimental Outcomes

**Figure 10:**
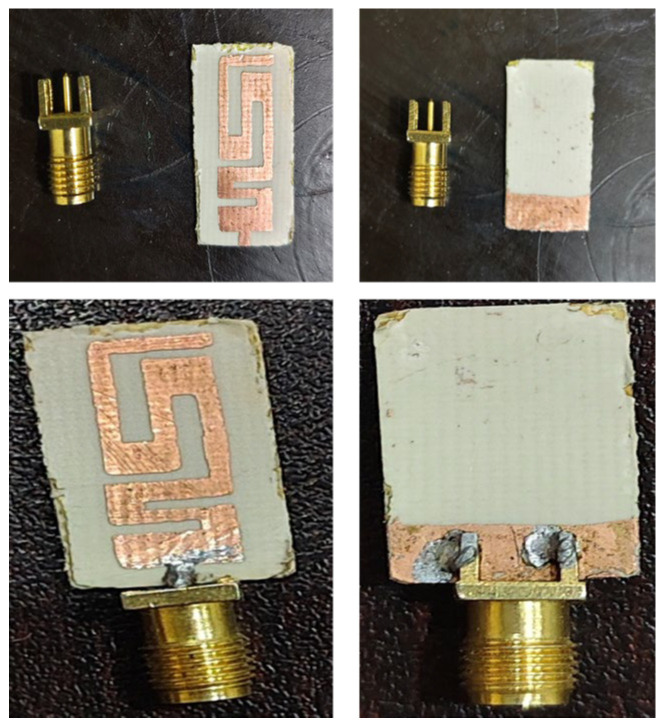
The fabricated prototype.

After the electromagnetic simulations were done, the optimized antenna was manufactured on a semi-flexible Arlon AD 450 substrate. The man-made prototype was then tested against the numerical findings in order to validate them. The structure that is fabricated is shown in Figure 10 and is excited using a microstrip feedline terminated by a Sub-Miniature Version-A (SMA) connector. SMA connectors are used in small-size RF systems because of their low loss and high frequency range with high broadband compatibility and good impedance matching.

### 3.1. Reflection Coefficient in Free Space

**Figure 11:**
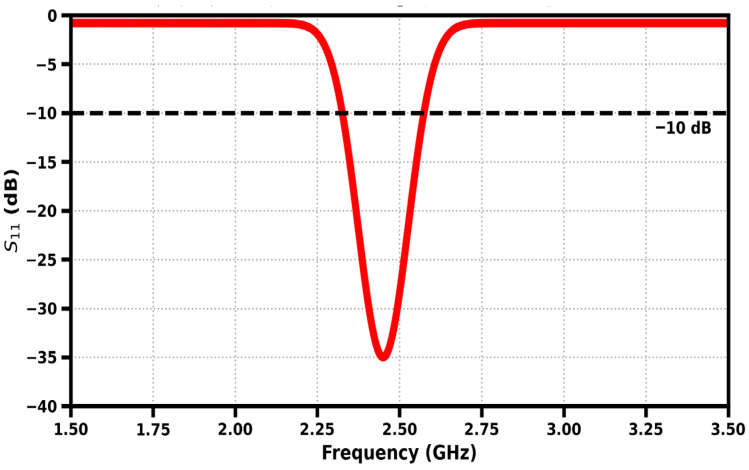
Reflection coefficient of the proposed antenna.

The simulated return loss response of the finalized meandered implantable antenna shows that the antenna has a well-matched resonance at 2.45 GHz with a minimum S_11_ of about −35 dB, which indicates efficient power transfer with minimum reflection. The geometry of the meander vastly improves the electrical length in the small footprint, making it possible to allow resonance in the ISM band as shown in Figure 11. The −10 dB impedance bandwidth of ~250-MHz (2.33–2.58 GHz) results in consistent detuning tolerance when different tissue loading and placement are used, which validates the design as appropriate for biomedical in-body communication.

**Figure 12:**
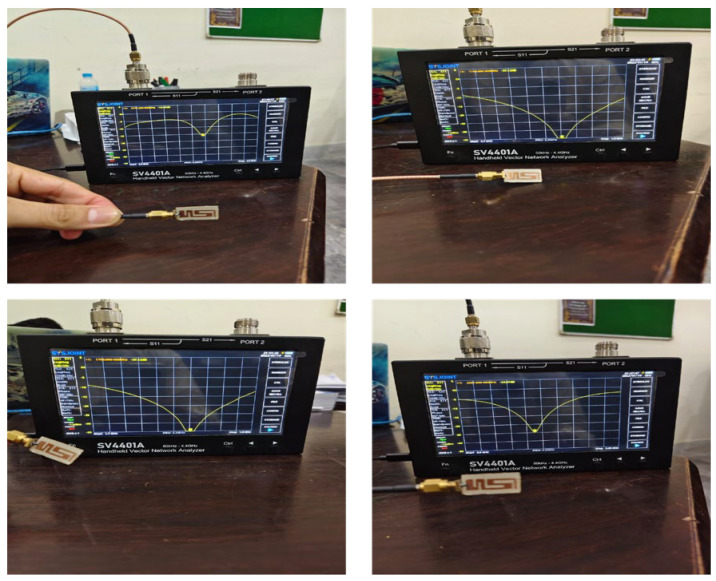
A measurement setup for S_11_ parameters of the proposed antenna.

Figure 12 shows a measurement setup for S_11_ parameter measurement of the proposed antenna in free space, i.e., without a phantom. A small, portable and cost-effective two-port Vector Network Analyzer S4401A manufactured by Chelegance from Shenzhen China was used for this purpose. It has a measurement range of 50 kHz to 4.4 GHz.

**Figure 13:**
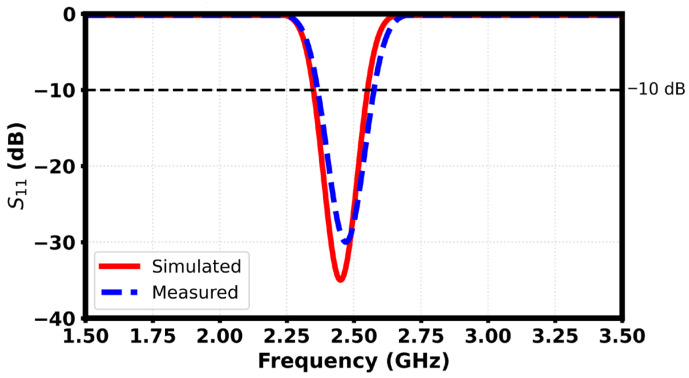
Reflection coefficient of the fabricated and simulated antenna.

Figure 13 is the comparison of the simulated and measured S_11_ characteristics of the proposed antenna. A good agreement is noted between both results, with the return loss consistently being less than −10 dB over the operating band. The small change in resonance that is apparent in the measured response, can be explained by tolerances in fabrication, including any deviation in dimensional features introduced in the milling of the PCB. Further variations can be caused by the inconsistencies of soldering and the thermal effect when the SMA connector is attached, which can cause a slight change in the feed and ground interface.

### 3.2. Radiation Pattern in Free Space

**Figure 14:**
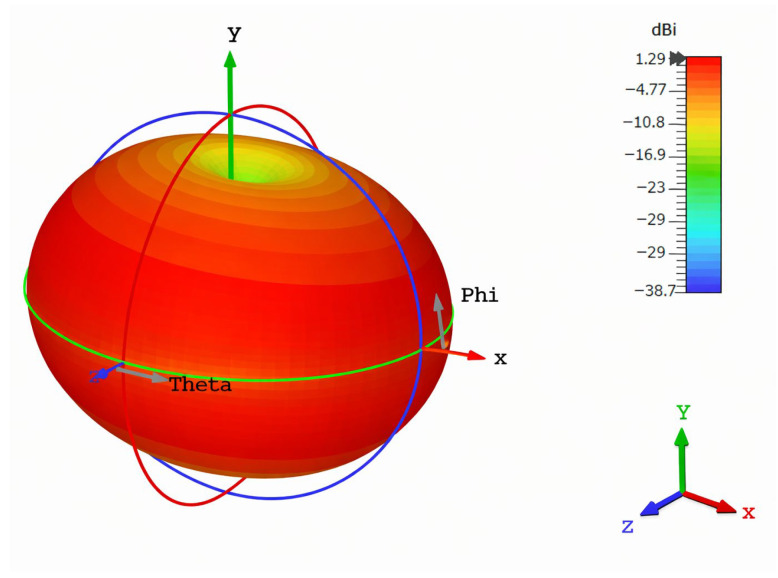
D radiation pattern of proposed antenna at 2.45 GHz.

Figure 14 shows the simulation of the three-dimensional far-field radiation pattern of the proposed antenna in spherical coordinates to identify the angular distribution of the realized gain. The radiation features are given in terms of the θ and φ plane sections, with the color-coded scale giving the gain variation in dBi. A dominant radiation lobe is detected in the broadside direction, whereas the overall pattern is smooth and well distributed, suggesting stable radiation behavior without a lot of distortion. The lack of strong nulls and quasi-omnidirectional performance in the principal planes make the antenna especially suitable for wearable and applications using RFD (radio frequency identification) technology where random antenna orientation and movement are common. Also, the antenna is suitable for implantable applications in which the antenna orientation as well as the tissue conditions around the antenna are inherently unpredictable. Such radiation characteristics provide stable electromagnetic coupling with the external readers under implant rotation or body motion, hence increasing the reliability of the link in lossy biological environment. The moderate directivity and broadside radiation are useful for efficient power transmission and for avoiding excessive localized deposition of energy, which is useful for meeting particular absorption rate (SAR) limitations. Additionally, the polarization behavior is often stable across the spectrum of operation, thereby reducing the mismatch losses and improving the robustness of the communication, which is essential for implantable medical devices that require continuous and reliable data transfer and typically use very low power. The radiation performance shows good spatial coverage and uniform polarization characteristics over the operating bandwidth, which ensures reliable communication in practical deployment situations.

The dimensions of the ground plane are 7 × 12 mm, which, at 2.4 GHz, is the electrical size:

$$\lambda_{0}=\frac{c}{f}=0.125m$$

$$\frac{L_{g}}{\lambda_{0}}=0.096,\;\frac{W_{g}}{\lambda_{0}}=0.056$$

$$kL_{g}=2\pi \frac{L_{g}}{\lambda_{0}}\approx 0.60,\;kW_{g}=2\pi \frac{W_{g}}{\lambda_{0}}\approx 0.35$$

Thus, although physically small, the ground plane is far from electrically negligible, and together with the radiator, it may support a weakly directive mode of radiation, which is at resonance.

To find the value of the expected realized gain, we apply the following standard relation:

$$G_{real}\left(\theta ,\varphi \right)=\eta_{rad}(1-{\left|\Gamma \right|}^{2})D(\theta ,\;\varphi )$$

By using simulated results, we find the following:

$$G_{real,max,lin}\approx 1.3489$$

$$G_{real,max}\approx 10\log\left(1.3489\right)\approx 1.3\;dBi$$

It can be seen from this that the realized gain in this case is still positive at 2.4 GHz despite the compact ground plane, mainly because of the high radiation efficiency and extremely low mismatch loss (−35 dB).

**Figure 15:**
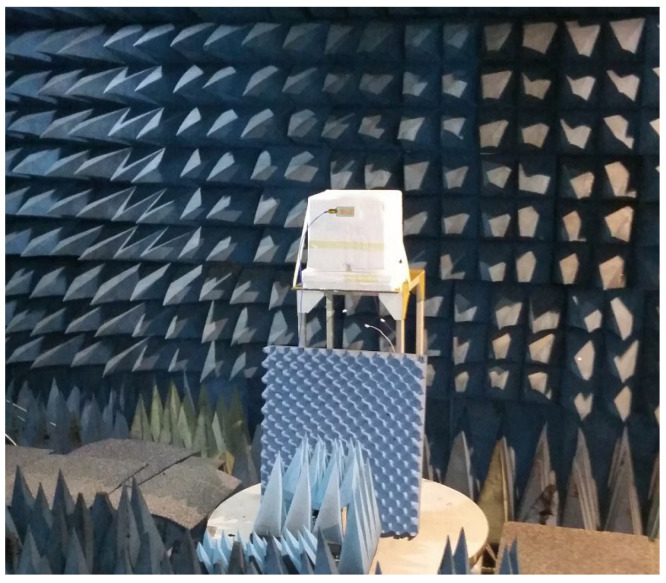
Measurement setup for radiation pattern.

An antenna measurement setup inside an anechoic chamber is shown in Figure 15. The walls and the floor of the chamber are covered with RF absorbers, which are pyramid-shaped foam structures whose purpose is to minimize the reflection of electromagnetic fields, thus presenting a free-space environment to accurately test the antennas. The antenna under test (AUT) is positioned on a stand made of non-reflective material at the center of the chamber and connected through coaxial cables.

**Figure 16:**
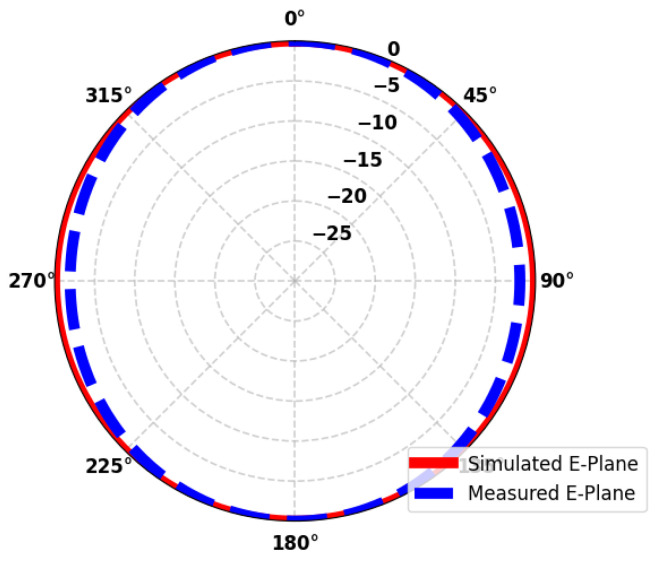
Illustration of simulated and measured E-field 2D radiation pattern.

Figure 16 depicts a plot of polar radiation patterns comparing the simulated and measured E-plane response of an antenna. The E-plane is the plane which includes the electric field vector and the direction of maximum radiation. The red solid curve is for the simulated data, whereas the blue dashed curve is for the measured results. The MW variables’ radial and angular axes are observed on the plot: the radial axis represents the gain (in dB), and the angular axis represents the azimuth angle (in a sequential plot: 0–360°). The close match between the two curves shows that there is good agreement between simulation and measurement, which indicates that the antenna shows the same characterization in terms of its directional performance in both theoretical and practical evaluations.

**Figure 17:**
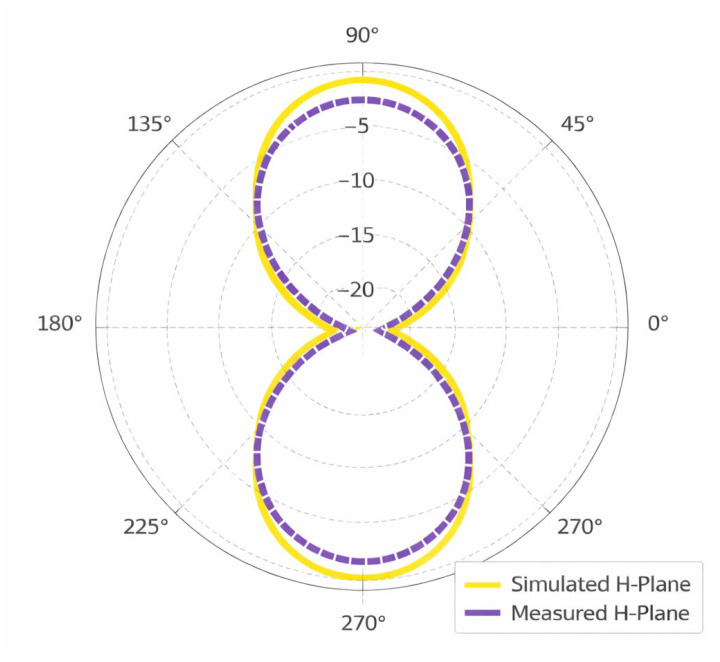
Illustration of simulated and measured H-field 2D radiation pattern.

Figure 17 shows a polar radiation pattern that shows the H-plane (magnetic field plane) performance of an antenna. The yellow solid line is the simulated H-plane, and the purple dashed one is the measured H-plane. The variation in the radiation along the azimuthal plane in the H-plane is usually called the H-plane pattern. In this case, the plot has a bi-directional or figure-eight shape, which is characteristic of linearly polarized antennas such as dipoles. The radial axis denotes the level of radiation in decibels (dB) and the angular scale denotes the azimuth angle (0° to 360° degrees). The closely similar simulated and measured curves show good agreement and validate the accuracy of the design of the antenna as well as the stable radiation factors of the antenna for both simulation and experimental measurements.

### 3.3. Efficiency and Gain in Free Space

**Figure 18:**
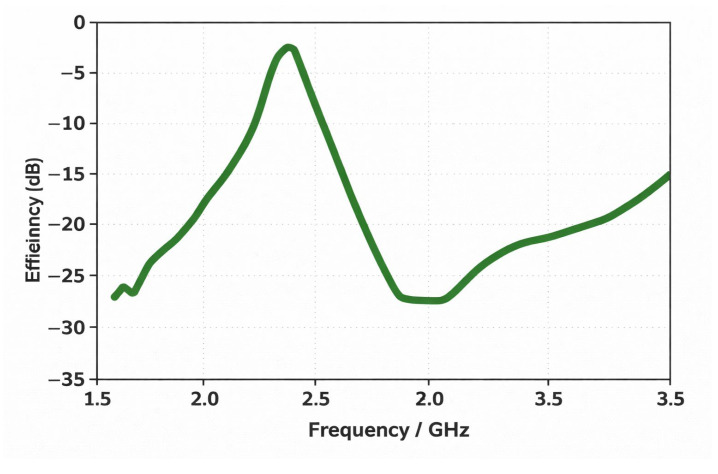
Antenna’s radiation efficiency.

The plot in Figure 18 is a function of the antenna efficiency (in dB) as a function of frequency (in GHz). The graph gives an idea of the varying efficiency of the antenna from 1.5 to 3.5 GHz. A special maximum of about 79% in efficiency is seen at around 2.45 GHz, the resonant frequency of the antenna; at this frequency, the antenna works very well, with minimal loss of power. Efficiency drops off considerably outside of this range, especially above 3.0 GHz and below 2.0 GHz, showing diminished radiation performance.

**Figure 19:**
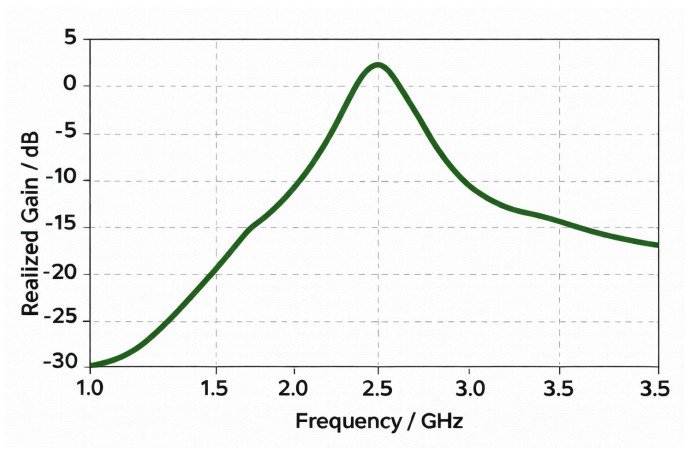
Antenna’s gain profile.

Figure 19 shows that at 2.45 GHz, the antenna has the peak value of the realized gain close to 1.29 dBi, which is the maximum of the radiation efficiency in the measured frequency range of 1~3.5 GHz. This enhancement in gain may be explained by the best impedance matching and low ohmic and dielectric losses at resonance frequency. The transition approach shows a progressively increasing gain from about −20 dB around 1 GHz that implies that the radiating structure tends to be more efficient as the frequency approaches the design band. Beyond 2.45 GHz, the gain drops off significantly and implies detuning effects, low radiation efficiency and potential mismatch of the antenna and feed network. Observed behavior confirms that the antenna is most effective for ISM-band application around the center frequency at 2.45 GHz, where maximum realizable radiated power is obtained.

### 3.4. Radiation Pattern and Antenna’s Gain Profile in a Surrounding Environment

**Figure 20:**
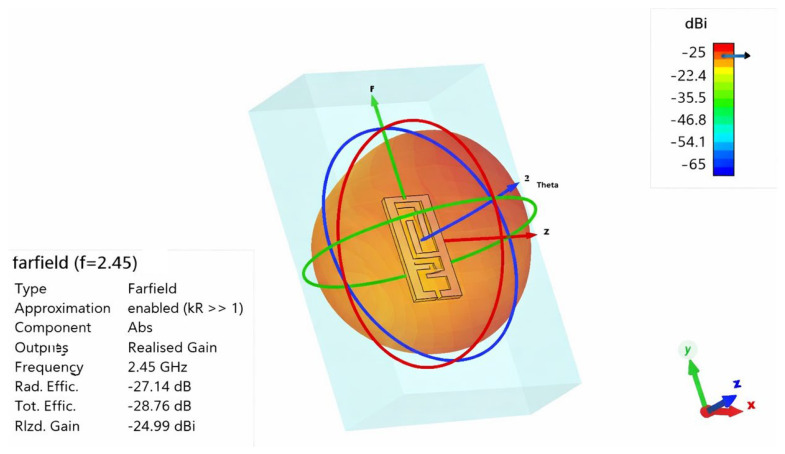
D radiation pattern of proposed antenna at 2.45 GHz inside the phantom.

The simulated far-field radiation performance of the proposed antenna inside a tissue-mimicking phantom at 2.45 GHz shown in Figure 20 shows the leading degradation due to dielectric loading and absorption losses. The realized gain is about −24.99 dBi and the radiation efficiency is about −27.14 dB (approx. 1.93%), as a reflection of the high level of attenuation that was introduced by the lossy biological medium. The total is further diminished because of mismatch losses and conductive losses. Despite such reduction, the radiation pattern maintains its stability and well-defined nature that denotes that the antenna maintains consistent radiative behavior in the phantom environment. This level of performance is in agreement with reported implantable antennas in biomedical implant applications operating in high-permittivity and -conductivity biological tissues and validates the practicality of the design of an implantable antenna for biomedical applications.

To check the implantable performance, the optimized antenna (without changing its physical dimensions) is placed inside a tissue-equivalent lossy phantom. Due to the dielectric loading and conductive losses that are introduced by the surrounding medium, a shift in resonance and deterioration of radiation efficiency and of realized gain were noticed. These effects are physically consistent with the operation of implantable antennas and result from the high permittivity and high conductivity of biological tissues.

**Figure 21:**
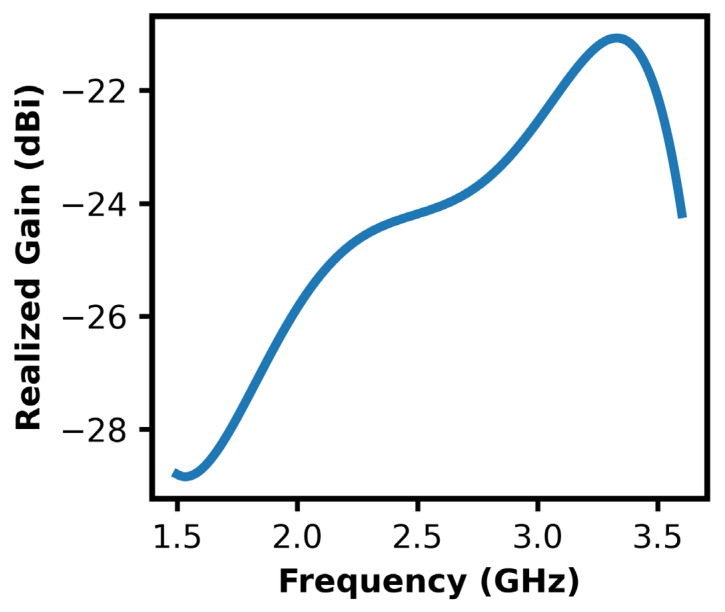
Antenna’s gain profile inside phantom.

The simulated realized gain of the proposed antenna embedded inside the tissue-mimicking phantom is shown in Figure 21. As can be expected, there is a significant effect on the radiation characteristics because of the presence of the lossy medium, due to dielectric loading and the existence of absorption losses. Compared to free space, the gain is attenuated in the entire frequency range, since a portion of the radiated power is dissipated in the high-permittivity conductive phantom material. Nevertheless, the antenna has a positive realized gain, with a maximum about 2.45 GHz, which confirms that the design is radiatively efficient regardless of the real implantation condition. The observed decreased gain is consistent with theoretical expectations of implantable antennas functioning in high-loss biological environments, while the maintained positive gain shows the suitability of the proposed structure to achieve reliable in-body and near-body wireless communication.

### 3.5. Gain Analysis and Electrical-Size Considerations

The free-space gain of 1.29 dBi at 2.45 GHz is determined here on the basis of electromagnetic analysis in order to make its physical consistency with the dimensions of the antenna (12 × 22 × 0.787 mm^3^) clear. The classical relation between the aperture

(7) $$G\approx \frac{4\pi A}{\lambda^{2}}$$

describes the theoretical directivity of an ideal uniformly illuminated planar aperture whose effective aperture is strictly equal to its physical projected area. This formulation is appropriate for canonical aperture antennas such as horns or reflectors, where radiation is governed by a well-defined aperture distribution. However, it does not directly describe compact resonant current-driven radiators.

The proposed antenna is a three-dimensional meander-line resonant structure, where the radiation mechanism is governed by the distribution mode of current but not the uniform aperture illumination structure. For general radiating structures, the effective aperture is defined as

(8) $$A_{eff}=\frac{\lambda^{2}G}{4\pi }$$

which depends on the far-field radiation pattern, which is not limited to the geometric footprint. So, at 2.45 GHz, the free-space wavelength is about 122 mm and the electrical dimensions of the antenna are about 0.10λ × 0.18λ. This size falls nowhere within the extremely electrically small regime.

For further determination of physical plausibility, the electrical size parameter $k_{a}$ can be taken into consideration where k=2πλ and a is the radius of the smallest possible circumscribing sphere. For the present geometry, a≈11 mm giving, $k_{a}=0.57$. The Chu–Harrington limit shows that severe restrictions on radiation come mostly from $k_{a}\ll 1$. Since our $k_{a}$ is in the order of 0.5, the antenna does not run in the deeply sub-wavelength area, where profoundly strong fundamental limitations to the radiation efficiency or obtainable directivity strongly constrain the antenna. The actual reported free-space directivity is therefore in agreement with the small-antenna theory.

The value of 1.29 dBi is the simulated free-space directivity based on a full-wave electromagnetic solver and verified radiation boundary conditions and mesh convergence. The antenna is a fully passive antenna. With the inclusion of radiation efficiency (that is about 79%), the realized gain is as follows:

(9) $$G_{realized}=D+10{log}_{10}\left(\eta \right)$$

which gives the same performance as the simulated response.

When placed in a tissue-equivalent lossy phantom, an appreciable fall in gain is found owing to the dielectric loading and conductive absorption losses, providing further evidence that the solver configuration does not artificially increase radiation characteristics.

Compact resonant radiators of similar electrical size often have free-space gains of the order of 0–3 dBi, as shown in Table 2. Therefore, the reported performance is consistent with the theory of electro-magnetic, electrical-size considerations and known compact antenna behavior.

### 3.6. Surface Current Distribution

**Figure 22:**
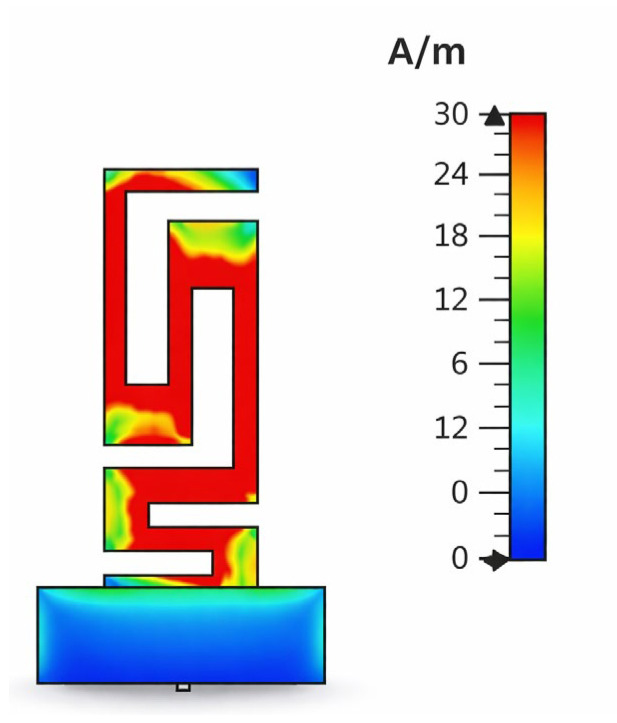
Distribution of surface current at 2.45 GHz.

The surface current distribution at the resonant frequency shows in Figure 22 that most of the excited current is concentrated at the meandered radiating strip, which proves its dominant position in radiation. The current peaks, which are visualized in red areas, show strong electromagnetic activity along the top and middle part of the serpentine conductor, which has validated the efficient extension of the electrical path created by the compact folded geometry. Reduced current density towards the feed and ground plane is representative of the expected transitions between radiation and current return at the ground plane acting mostly as a current sink and not as a radiator. The occurrence of weaker currents (blue–green) at bending-points is the result of locally occurring impedance variations created by each segment. Overall, the distribution validates the antenna’s ability to provide resonance by having an efficient excitation of the extended current path, without causing large physical dimensions—which is considered to validate this antenna for its implantable applications.

## 4. Modeling of Electrical Equivalent Circuit

**Figure 23:**
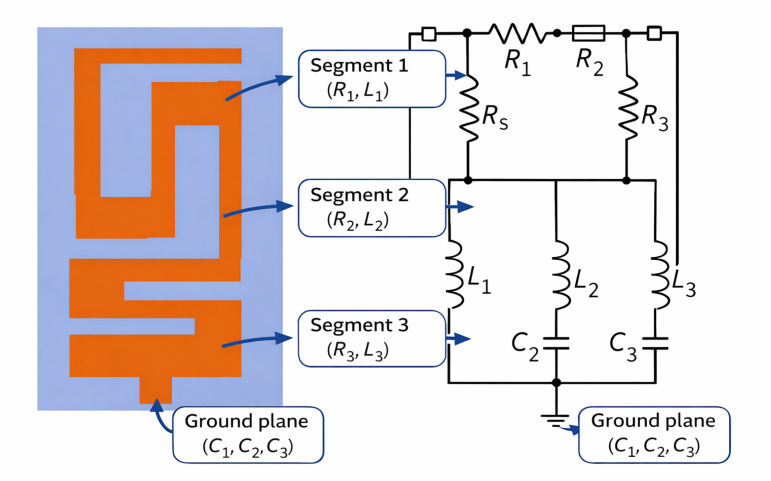
Equivalent circuit for meandered patch antenna geometry.

In order to provide physical insight into the electromagnetic operation of the proposed meandered patch antenna, an equivalent lumped-element model for the proposed patch antenna was formulated at the operating frequency of 2.45 GHz. The model is based on three cascaded resistive–inductive (R-L) branches which represent the meandered current path, representing an electrically different part of the conductor. Segment 1, Segment 2 and Segment 3 in the antenna layout are thus mapped to R_1_–L_1_, R_2_–L_2_ and R_3_–L_3_, respectively, as shown in Figure 23. These inductances are used to capture the stored magnetic energy in the long path of the folded current path, while the series resistances are used to account for the conductor transmission loss, loss in insulating material and radiation damping inherent to each of the sections.

In parallel to these inductive branches come the fringing electric fields between the patch and ground plane, which are modeled by means of three shunt capacitors, C_1_, C_2_ and C_3_. These lumped capacitive elements model the distributed capacitive coupling that exists along the meandered edges that is known to be dominant in the stored electric energy in electrically small patches. The combination of the branches in the RLC circuit leads to a single frequency resonance and, when properly tuned, an input impedance close to 50 ohms at 2.45 GHz. The agreement between measured equivalent-circuit reflection coefficients (S_11_) and the agreement verifies the validity of the circuit used to represent the main resonance mechanism and its capability for reproducing well the distributed-to-lumped transformation of the electromagnetic behavior of the antennas. This model therefore allows an efficient way to predict impedance and bandwidth characteristics without having to run repeated full-wave simulations.

To achieve an analytical interpretation of the electromagnetic behavior of the proposed meandered microstrip antenna, the structure can be modeled by a lumped-element equivalent network and comprises three parallel RLC branches, each of which represents one electrically significant segment of the meandered conductor. Each branch consists of a series resistance R_i_, inductance L_i_ and capacitor C_i_ in shunt for i = 1, 2, 3. Another modeling feed resistance R_s_, a conductor, a conductor and a dielectric loss, is close to the input port. The impedance of ith branch is given by

(10) $$Z_{i}\left(\omega \right)=R_{i}+j\omega L_{i}+\frac{1}{j\omega C_{i}}$$

If, at resonance, $\omega_{0}=2\pi f_{0}$, the reactance cancels. Thus, each branch reduces to purely resistive. The input resistance presented to the feed includes the series feed loss:

(11) $$Z_{i}\left(\omega_{0}\right)=R_{eq}+R_{s}$$

A good impedance match is achieved at $Z_{i}\left(\omega_{0}\right)=50\;\Omega$. For each branch, resonance occurs at

(12) $$f_{0}=\frac{1}{2\pi \sqrt{L_{i}C_{i}}}$$

ensuring that all three RLC branches collectively support a single dominant resonance near 2.45 GHz.

The quality factor is given by

(13) $$Q_{i}=\frac{\omega_{0}L_{i}}{R_{i}}$$

The measured bandwidth at −10 dB is given by

(14) $$BW=\frac{f_{0}}{Q_{eq}}$$

Table 3 shows the estimated segment-wise length fractions and corresponding lumped inductance and capacitance values calculated from the above formulae and used in the equivalent-circuit model of the antenna.

**Figure 24:**
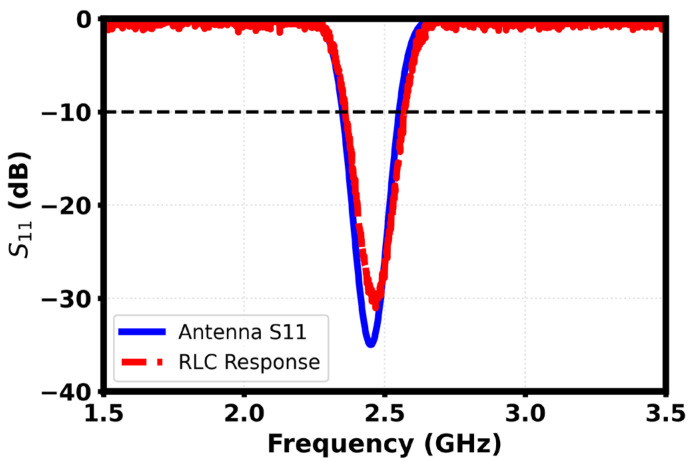
Comparison of the simulated S_11_ of the meandered antenna and the equivalent RLC circuit.

Corresponding values of R are given by $R_{1}\approx 120\;\Omega ,\;{\;R}_{2}\approx 110\;\Omega \;and\;R_{3}\approx 90\;\Omega$. At the resonant level of 2.45 GHz, the inductive and capacitive reactance of the two branches of the RLC are identical, and the three lossy resonators collapse into a common real resistance, which is matched to the feed. The resultant equivalent impedance and bandwidth accurately reproduce the observed return loss data obtained for the antenna when the RLC network is used as a model for the antenna geometry at the electromagnetic scale. Thus, the RLC network in its simplest form satisfies the model validation issue that models for antenna systems must be compact and must be physically meaningful models of the corresponding part of the antenna geometry. Figure 24 shows a comparison of the simulated S_11_ of the meandered antenna and the equivalent RLC circuit response.

## 5. SAR Analysis

Specific absorption rate (SAR) is an important parameter in the design and testing of on-body and in-body wireless communication devices because it is a measure of the rate of absorption of electromagnetic energy by human tissue. When a radiating element is operated in close proximity to the body, then a portion of the emitted electromagnetic field will penetrate into the biological tissues, producing energy depositions with potentially undesirable or even destructive thermal effects or disruption of normal physiological processes. In order to evaluate and reduce these risks, SAR values are mathematically calculated and tested to allow designers to predict energy exposure levels and make sure the radiation exposure is within an acceptable safety level. According to the IEEE C95.1 v 1999 safety standard, the maximum allowed SAR is 1.6 W/kg equivalent to 1 g tissue average power, which is a regulatory limit designed to prevent the user from being thermally overloaded and to ensure the safe operation of wearable and body-mounted devices. Compliance with SAR requirements is therefore essential for on-body antennas in order to give assurances that the level of the absorbed energy is kept minimal and that the antennas do not infringe upon the health of the user.

(15) $$SAR=\frac{\sigma E^{2}}{2\rho }$$

**Figure 25:**
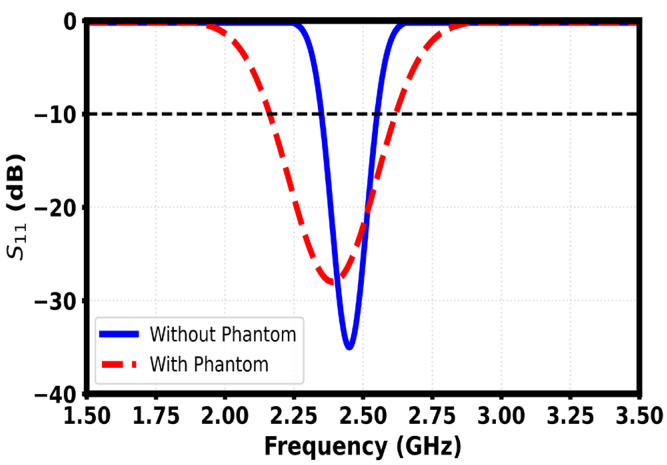
Reflection coefficient with and without phantom.

Simulated return loss characteristics show a distinct difference between free-space performance and loading performance caused by the human tissue phantom as shown in Figure 23. Without the phantom, the antenna shows a deep resonance around 2.45 GHz with an S_11_ minimum below −30 dB, which is great impedance matching and also a good indication of efficient power transfer. When the position of the phantom is set close to it, the resonant dip tends to descend in frequency and become shallower, with the minimum magnitude decreasing to about −25 dB. This behavior is a result of the combination of the dielectric loading, absorption losses and detuning resulting from the high permittivity and conductivity of biological tissue. The wider bandwidth under phantom loading implies increased damping and lowering of the quality factor, which is typical for on-body antennas. Overall, the response verifies that although the aspect of body proximity could have degraded the matching performance, the antenna still maintains adequate resonance within the band of 2.4 GHz, known as the ISM band, the adequacy of which would validate the antenna for wearable and implant adjacent wireless applications, see Figure 24 and Figure 25.

In order to experimentally validate the antenna performance for in-body loading, the prototype would be immersed inside a liquid tissue equivalent phantom with the formulation developed to simulate the dielectric properties of human soft tissue at 2.45 MHz (2.45 GHz). In order to experimentally describe the antenna performance in a biologically relevant environment, an agar-based tissue mimicking phantom was made in order to mimic the dielectric properties of human muscle tissue. The formulation was designed to have a relative permittivity of ~50 and a conductivity similar to biological muscle tissue in the frequency range in which it was to have a potential. Table 4 shows the materials and composition per 100 mL.

An agar-based muscle-mimicking phantom was fabricated for emulating the dielectric properties of human muscle tissue for experimental evaluation of implantable and wearable antennas. About 80 mL deionized water was heated to 60–70 °C, and sodium chloride was dissolved gradually and continuously with stirring until a homogeneous ionic solution was obtained. Agar powder was then introduced slowly, while keeping constant stirring and heating until the complete dissolution of the powder was attained, resulting in a clear to slightly turbid mixture, avoiding boiling to prevent bubble formation and nonuniformities in the concentration. Glycerol was afterwards included for the precise adjustment of the effective permittivity, while the mixture was stirred together well for uniformity of dispersion. Deionized water was added as needed to make up for evaporation and to bring the total volume to 100 mL. The warm solution was poured in the desired mold and left to solidify by cooling to room temperature or refrigeration for faster solidification, resulting in mechanically stable gel suitable for repeated measurements. The dielectric properties of the phantom were controlled by adjusting the concentrations of NaCl and glycerol with the outcome of relative permittivity (=45–55) being achieved by use of 2–3 wt% agar and 0.5–0.9 wt% NaCl and conductivity values of between 0.4 and 1.0 S/m as those of human muscle tissue. This formulation allows a reproducible, inexpensive and tissue-equivalent medium for realistic electromagnetic characterization of implantable and wearable systems for use as antenna systems.

The antenna was connected to a calibrated vector network analyzer (VNA) with a low-loss coaxial feed to minimize the measurement distortion. The measured S_11_ response shows a definite shift in resonant frequency upon submersion in the phantom, in good agreement with the predicted detuning behavior through electromagnetic simulations. This way, the experimental setup captures the electrical scenario occurring during on-body operation, similar to real-world scenarios, and it allows realistic evaluation concerning impedance matching, bandwidth reduction, and material absorption objects.

**Figure 26:**
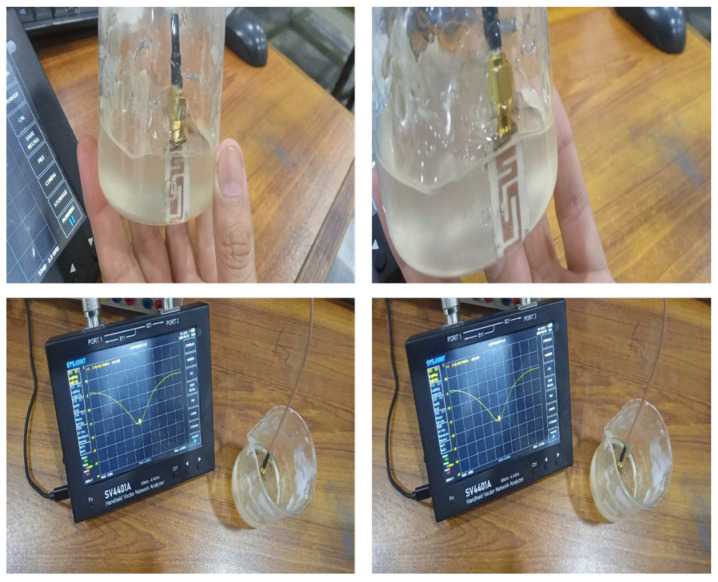
Measurement setup for reflection coefficient with phantom.

**Figure 27:**
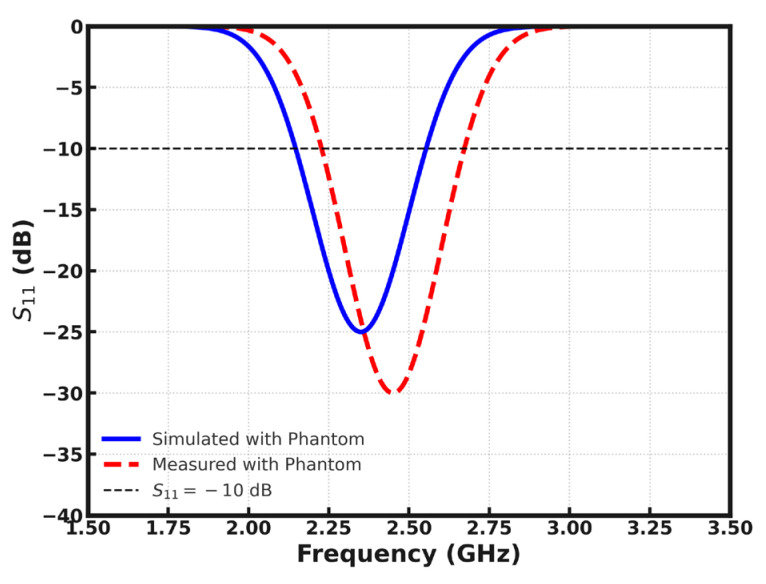
Comparison of simulated and measured reflection coefficient with phantom.

Figure 26 shows the distribution of the simulated electromagnetic field of the proposed antenna inside the multilayer losses, i.e., in a biological tissue environment. The color contour map shows the spatial variation of the intensity of the electric field—the high field confinement in the vicinity of the radiating elements and a slow decay into the surrounding layers. This behavior represents good coupling of the antenna to the surrounding medium coupled with low diffraction penetrating into the medium, which is essential for implantable and wearable applications. The multilayer configuration captures the effect of surrounding tissues on the antenna performance, which demonstrates control of the near-field interaction for the antenna and lower sensitivity to the environmental loading. Overall, the field distribution validates the validity of the antenna in terms of efficient radiation characteristics and the safe operation and stable performance in the complex tissue-mimicking environment, see Figure 27.

**Figure 28:**
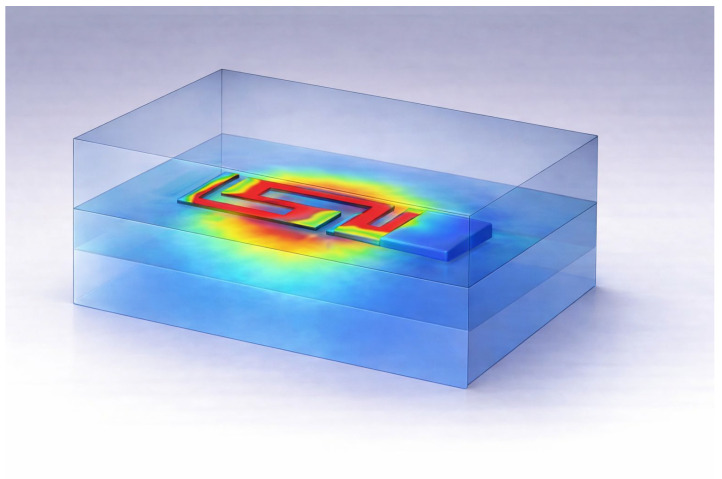
Simulated electric field distribution of the proposed antenna embedded within a muscle-equivalent phantom.

**Figure 29:**
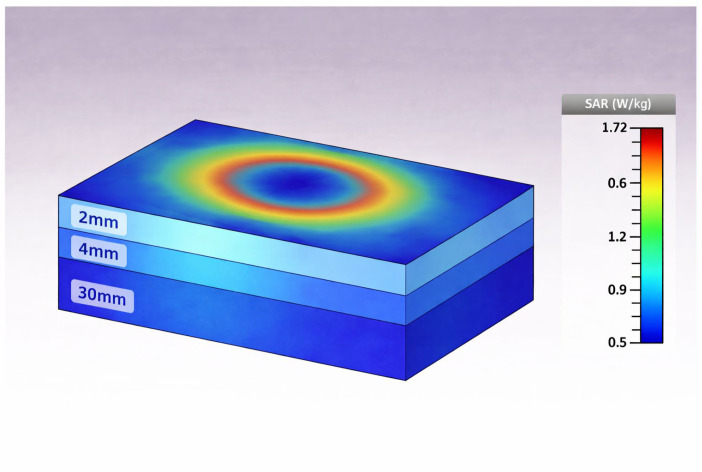
Simulated SAR distribution (W/kg) within a multilayer muscle-equivalent phantom.

The distribution of the simulated specific absorption rate (SAR) in a multilayer tissue-equivalent model when the antenna is situated at the design frequency is shown in Figure 28. The color map shows the spatial variation in the SAR in units of W/kg, where the highest localized absorption is seen in the area nearest to the antenna and a smooth decay occurs away from the radiating element in the tissue layers. The evaluation of the SAR is done based on a 10 g tissue-averaging mass, as per international safety guidelines such as the standards of the IEEE and ICNIRP. The maximum 10 g averaged SAR value, however, remains within the prescribed safety limits and therefore indicates controlled electromagnetic energy deposition, indicating that no excessive localized heating occurs. This behavior ensures that the resulting design of the antenna is the optimal balance between radiation performance, biological safety, and carbon efficiency for wearable and implantable applications where upholding the standards set by the SAR regulations is an important requirement, see Figure 29.

## 6. Results and Discussion

The proposed miniaturized meander-line antenna was proposed as a result of a systematic design development that aimed at resonating in the target RFID band with an acceptable appendage size that can be implanted or worn. The original reference geometry had a resonance frequency slightly different from the target one because of the small size of the radiator (electrically). A meander-line arrangement was added to deal with this and in effect enhance the electric length without increasing the size. This change brought the resonant frequency much lower, the path length of the surface current longer, and the effective inductive loading higher. The distributed capacitance between neighboring traces was then optimized by proper choice of meander spacing and density of segments to get the desired resonance tuning and allow better impedance matching. It was possible to do final fine tuning with an adjustment of feed structure and ground structure, which stabilized the response of the impedance and reduced sensitivity to fabrication capabilities and loading of the environment. With the emulated phantom of using tissues, the antenna showed the anticipated down-shift in bandwidth and broadening as its effective permittivity and dielectric loss increase in the surrounding media was accompanied with steady functioning at the target frequency.

An analogous lumped-element model was created to model the input impedance near the fundamental resonance so that circuit-level insight can be gained into the antenna behavior and system-level assimilation can take place in a reasonable amount of time. The radiator is electrically small and, therefore, the antenna response can be well modeled by a single resonant mode, which was modeled using a series topology based on the RLC model with less error. Here, the individual contribution of the meandered current path to the equivalent inductance is the largest, with inter-segment coupling and radiator-to-ground interaction being modeled instead by an effective capacitance. The resistive element considers the radiation and loss effects, which are very extreme, especially when the tissue is under loading. The circuit parameters were determined by comparing the simulated and measured impedance responses to determine the resonance condition, and from the corresponding bandwidth, we estimated the loaded quality factor. The resulting equivalent circuit has a close correspondence with the full-wave reflection coefficient characteristics, which validates the idea that the circuit can capture the most dominant electromagnetic behavior of the proposed antenna.

The proposed antenna was comprehensively analyzed in two different scenarios, including the free-space condition and in a tissue-mimicking phantom to simulate real-life biomedical scenarios. In free space, an antenna with stable impedance matching characteristics as designed, satisfactory return loss, VSWR < 2 and well-defined radiation characteristics has been obtained. The realized gain and radiation efficiency was very good despite this, confirming that impedance matching was correct, and the power was being radiated well. The radiation pattern showed stable broadside characteristics with acceptable directivity and negligible distortion in order to validate the suitability of the design for biomedical wireless applications.

To check the in-body applicability, the antenna was then analyzed in-body in a multilayer tissue-equivalent phantom. As expected, the effect of high-permittivity and lossy biological media on the electromagnetic behavior of the antenna is discussed. A small resonance shift was observed as a result of the dielectric loading effects; a significant degradation was observed in both realized gain and radiation efficiency. This decrease is attributed mainly to an increase in absorption and conductive losses in the surrounding tissues, which attenuates the radiated fields and decreases the effective power transmission. In addition, near-field interaction between the antenna and lossy medium was responsible for pattern distortion and diminished radiation intensity.

Despite the degradation in the gain and efficiency in phantom conditions, the required impedance matching and the operational bandwidth of the antenna were acceptable from the target and aimed frequency bands. The performance is, however, still sufficient for short-range biomedical communication links, such as implantable and wearable systems, in which reliable communication connections are needed under highly lossy propagation environments. These results validate the use of the proposed antenna in ideal and realistic biomedical environments for the first time, which shows the potential that the antenna may have for practical applications in the medical field for healthcare and implantable communications.

The measured and simulated reflection coefficients show good agreement, which confirms the validity of the design technique and fabrication accuracy. The resonance of the antenna in free space occurs at the target frequency within the −10 dB bandwidth of the impedance that can appropriately support the target RFID operation. When incorporated within or put close to the muscle-equivalent phantom, the resonance rises to a reduced frequency, as expected, and the acceptable impedance matches throughout the operating range. Small differences between simulation and measurements are ascribed to fabrication errors, connector errors and phantom error in the dielectric properties. These factors notwithstanding, the antenna is highly compatible around the target frequency, which proves its viability when it comes to practical implantation and wearable tasks.

The radiation properties were investigated in the simulated and measured patterns of the main planes. In open areas, the antenna can display a constant radiation pattern that is maintained by its small size geometry and bottom arrangement. The radiation level is cut at lower frequencies when the tissue-equivalent loading and the near-field interaction are more dominant through absorption in the lossy material but the pattern-shape of the total pattern still is similar to what the simulation predicted. The similarities between the simulated and measured trends imply that the antenna has predictable radiation behavior across the operational band, and therefore the antenna is important for effective RFID communication under different orientations.

The radiation efficiency and the gain attained by the antenna had been determined over the frequency range of interest. The electrically small size of the antenna allows achieving a peak realized gain and radiation efficiency in medium and high frequencies, in a free-space environment. Both gain and efficiency go down when it is in the tissue-equivalent environment because the dielectric loss and power absorption by the surrounding medium increases. However, the maximum gain is still concentrated at the target operating frequency, which illustrates that the antenna is also well tuned when heavily loaded. The trends are also in line with the equal circuit model, where high loss resistance is associated with low radiated power and a wider range of response to impedance.

The issue of safety was considered with the help of specific absorption rate (SAR) analysis performed in the phantom of the muscle. The SAR distribution shows the concentrated energy absorption around the antenna surface and a rapid decadent distribution with depth, which is typical of near-field coupling in lossy biological material. The highest value of the SAR recorded under the given input power and averaging mass lies under the acceptable levels of safety concerns on a normalized scale of common RFID excitation. The depth-dependency of the SAR behavior also supports that the energy absorption is much reduced with the distance between the antenna and, thus, the proposed design, which is implantable and wearable, is valid.

In general, the findings show that the proposed miniaturized meander-line antenna can be used in practice with effective matching of impedance, stable radiation, an acceptable gain and efficiency when loaded with the tissue as an equivalent load, and that it can meet the safety requirement of SAR in operation. This compliance of the design strategy and experimental output in full-wave simulation and the equivalent circuit model reveals the accuracy and possible application of the antenna in practical implantable and wearable RFID communication networks.

## 7. Conclusions

This paper presented the design, circuit-level modeling, and experiment validation for a miniaturized meander-line antenna that is targeted for implantable and wearable RFI biomedical applications. The proposed antenna can offer stable impedance matching over the targeted operating band with a compact form factor, which is suitable to be integrated with actuated biomedical devices. The developed equivalent circuit model is able to capture the dominant resonance behavior and correlates well with the full-wave simulated impedance response, ensuring the validity of the analytical approach. Both simulation and the measured results show correct radiation performance and thus confirm the reliability of the design methodology. The evaluation of the antenna was tested in free space and inside a tissue-mimicking phantom to simulate the in-body electromagnetic environment. While dielectric loading and conductive losses of biological tissues were responsible for the reduction in realized gain and radiation efficiency of the artificial antenna, acceptable impedance matching and bandwidth as well as stable radiation characteristics of the artificial antenna within the desired frequency band were achieved. In addition, the values of the specific absorption rate (SAR) were at established limits of safety, demonstrating adhesion to biomedical regulatory standards. Overall, the obtained results support the suitability of the proposed antenna for practical implantable and wearable healthcare systems working under realistic electromagnetic conditions with RFI (Radio Frequency Interference) and based on RF (radio frequency).

## Figures and Tables

**Table 1:** Frequency bands for medical and implantable communication systems [8].

Service/Technology	Allocated Frequency Band(s)
Medical Implant Communication Service (MICS)	402–405 MHz
Medical Device Radio Communications Service (Med Radio)	401–406 MHz, 413–419 MHz, 426–432 MHz, 438–444 MHz, 451–457 MHz
Industrial, Scientific, and Medical (ISM) Band	433.05–434.79 MHz, 902–928 MHz, 2400–2483.5 MHz, 5725–5850 MHz
Wireless Medical Telemetry Service (WMTS)	608–614 MHz, 1395–1400 MHz, 1427–1432 MHz
Ultra-Wideband (UWB)	3100–10,600 MHz
Medical Body Area Network (MBAN)	2360–2400 MHz

**Table 2:** Comparison of proposed antenna with previous studies.

Ref.	Size (mm^3^)	Frequency (GHz)	Substrate	BW (MHz)	Design Technique	Inside Tissue Gain (dBi)	Free Space Gain (dBi)
[27]	10 × 10 × 0.635	2.4	Rogers RT 5880	828	Meander Line	−17.3	-
[28]	15 × 15 × 15	2.4	Rogers RT 5880	-	Patch Antenna	−18.5	-
[29]	22 × 22 × 0.635	0.402	Rogers RT 6010	390	Planar Monopole	−26	-
[30]	32 × 7 × 0.5	2.464	PDMS	300	Planar	−15.18	-
[31]	17.2 × 14.4 × 0.254	2.4	Rogers RO 3010	190	Fractal Geometry	−17	-
[32]	11 × 19 × 1.25	2.4	Rogers RO 3210	100	PIFA Design	−11.8	-
[33]	6 × 6 × 2.54	2.45	Arlon AD 1000	200	Slotted Planar	−28	-
[34]	7 × 7 × 0.2	2.45	Rogers ULTRAM	420	Shorting Pin	−15	-
[35]	10 × 10 × 2.56	2.4	Rogers RO 3210	100	TARS	-	-
[10]	3 × 4 × 0.5	2.4	Rogers RT 6010	475	Meander Line	−25.95	2.15
[36]	57 × 55 × 0.7	2.4	Taconic TLX (tm)	-	Slotted	-	5.09
[37]	3.48 × 4.56 × 0.5	20	FR4	940	Notch Based	−27	5.5
[38]	15 × 15 × 1.27	0.915	Rogers RO 3010	97	Slotted	−27	2.15
[39]	7 × 6 × 0.5	2.45	Rogers RT 6010	100	Slotted	−26.4	2
[40]	11 × 11 × 1.27	0.915	Rogers RO 3010	-	Slotted	−29	-
[41]	60 × 80 × 1.5	2.4	SVR-PLAD		Slotted	-	−1
[42]	7 × 7 × 0.254	0.915	Rogers RT5880	220	Bear Shaped	−22	-
[43]	40 × 35 × 1.6	2.4	FR4	120	CDRA	-	2.01
This Work	12 × 22 × 0.787	2.45	Arlon AD 450	250	Meander Line	−24.99	1.29

**Table 3:** Lumped inductance and capacitance values for individual antenna segments.

Segment	Length Fraction	Lumped Inductance	Lumped Capacitance
1	~40%	$L_{1}\approx 0.21\;nH$	$C_{1}\approx 3.4\;pF$
2	~35%	$L_{2}\approx 0.18\;nH$	$C_{2}\approx 3.0\;pF$
3	~25%	$L_{3}\approx 0.14\;nH$	$C_{3}\approx 2.1\;pF$

**Table 4:** Materials and composition (per 100 mL).

Component	Function	Quantity
Agar powder	Gelling agent and structural matrix	2.0–3.0 g
Sodium chloride (NaCl)	Conductivity control	0.4–0.9 g
Deionized (DI) water	Base medium	Up to 100 mL
Glycerol	Dielectric constant tuning	5–10 mL

## Data Availability

No new data were created or analyzed in this study.
